# Impact of Insulin-Treated Compared to Non-Insulin-Treated Diabetes Mellitus on Outcome of Percutaneous Coronary Intervention with Drug-Coated Balloons versus Drug-Eluting Stents in De Novo Coronary Artery Disease: The Randomized BASKET-SMALL 2 Trial

**DOI:** 10.3390/jcdd10030119

**Published:** 2023-03-13

**Authors:** Julia Seeger, Jochen Wöhrle, Bruno Scheller, Ahmed Farah, Marc-Alexander Ohlow, Norman Mangner, Sven Möbius-Winkler, Daniel Weilenmann, Georg Stachel, Gregor Leibundgut, Peter Rickenbacher, Marco Cattaneo, Nicole Gilgen, Christoph Kaiser, Raban Jeger, on behalf of the BASKET-SMALL 2 Investigators

**Affiliations:** 1 Medical Campus Lake Constance, Department of Cardiology and Intensive Care, 88048 Friedrichshafen, Germany; 2 Clinical and Experimental Interventional Cardiology, University of Saarland, 66421 Homburg, Germany; 3 Knappschaftskrankenhaus, Klinikum Westfalen, 44143 Dortmund, Germany; 4 SRH Wald-Klinikum Gera, 07548 Gera, Germany; 5 Herzzentrum Dresden, Department of Internal Medicine and Cardiology, Technische Universität Dresden, 01062 Dresden, Germany; 6 Department of Cardiology, University Hospital Jena, 07747 Jena, Germany; 7 Cantonal Hospital St Gallen, 9007 St. Gallen, Switzerland; 8 Heart Center Leipzig, University Hospital, 04289 Leipzig, Germany; 9 Cantonal Hospital Baselland, 4410 Liestal, Switzerland; 10 University Hospital Basel, University of Basel, 4001 Basel, Switzerland; 11 Department of Cardiology, Cardiovascular Research Institute Basel (CRIB), University Hospital Basel, 4031 Basel, Switzerland; 12 Department of Cardiology, Triemli Hospital Zurich, 8063 Zurich, Switzerland

**Keywords:** drug-coated balloon, drug-eluting stent, target vessel revascularization, small vessel disease, diabetes mellitus

## Abstract

Background: We evaluated the outcome of PCI of de novo stenosis with drug-coated balloons (DCB) versus drug-eluting stents (DES) in patients with insulin-treated diabetes mellitus (ITDM) versus non-insulin-treated diabetes mellitus (NITDM). Methods: Patients were randomized in the BASKET-SMALL 2 trial to DCB or DES and followed over 3 years for MACE (cardiac death, non-fatal myocardial infarction [MI], and target vessel revascularization [TVR]). Outcome in the diabetic subgroup (*n* = 252) was analyzed with respect to ITDM or NITDM. Results: In NITDM patients (*n* = 157), rates of MACE (16.7% vs. 21.9%, hazard ratio [HR] 0.68, 95% confidence interval [CI] 0.29–1.58, *p* = 0.37), death, non-fatal MI, and TVR (8.4% vs. 14.5%, HR 0.30, 95% CI 0.09–1.03, *p* = 0.057) were similar between DCB and DES. In ITDM patients (*n* = 95), rates of MACE (DCB 23.4% vs. DES 22.7%, HR 1.12, 95% CI 0.46–2.74, *p* = 0.81), death, non-fatal MI, and TVR (10.1% vs. 15.7%, HR 0.64, 95% CI 0.18–2.27, *p* = 0.49) were similar between DCB and DES. TVR was significantly lower with DCB versus DES in all diabetic patients (HR 0.41, 95% CI 0.18–0.95, *p* = 0.038). Conclusions: DCB compared to DES for treatment of de novo coronary lesions in diabetic patients was associated with similar rates of MACE and numerically lower need for TVR both for ITDM and NITDM patients.

## 1. Introduction

Patients with diabetes mellitus and coronary artery disease often present with a combination of diffuse coronary lesions and small vessel disease associated with multiple comorbidities increasing the bleeding risk, which inadvertently impacts overall outcomes after percutaneous coronary intervention (PCI). Lesions in diabetic patients consist of lipid-laden plaques with thin fibrous capsules and high calcium content, which increases their vulnerability for rupture and repeat thromboembolic events. In addition, vascular resistance based on alterations of the molecular interaction in smooth muscle cells limits the action of anti-proliferative agents eluted by coronary stents [1]. Hence, treatment with drug-eluting stents (DES) may be associated with technical difficulties in stent delivery and expansion in those diffusely diseased calcified lesions in small vessels and is associated with an increased risk of stent thrombosis and the need for revascularization. 

For de novo lesions in small coronary arteries, several trials have shown similar results with the use of drug-coated balloons (DCB) compared to DES [2,3,4]. A possible advantage with the use of DCBs is an antirestenotic efficacy which is associated with the potential for late lumen enlargement in the absence of a metallic stent [5]. Since there is no permanent vascular implant, the risk of late or very late stent thrombosis is eliminated and the need for dual antiplatelet therapy (DAPT) in stable patients can be limited to 4 weeks [2], reducing the bleeding risk.

However, the risk of restenosis, myocardial infarction and stent thrombosis is increased in diabetic compared to non-diabetic patients, both for treatment with DES and DCB [6,7,8,9]. PCI with DES in patients with insulin-treated diabetes mellitus (ITDM) is associated with a significantly higher target lesion failure rate and higher need for revascularization compared to PCI in patients with non-insulin-treated diabetes mellitus (NITDM) [10,11,12,13,14]. Whether the outcome of PCI of de novo coronary artery disease using DCB or DES in patients with ITDM or NITDM is different has not been studied so far.

We analyzed the impact of insulin-treated compared to non-insulin-treated diabetes mellitus in patients undergoing PCI of a de novo lesion on the outcome of drug-coated balloons versus drug-eluting stents in the diabetic population of the randomized BASKET-SMALL 2 trial.

## 2. Materials and Methods

### 2.1. Study Design

BASKET-SMALL 2 [15] is an investigator-initiated, randomized, open-label, non-inferiority trial demonstrating a similar efficacy and safety for DCB compared with DES for 3 years in 758 patients with de novo lesions in coronary vessels < 3 mm [2,3]. This subgroup analysis compares the efficacy and safety within 1, 2 and 3 years between patients with NITDM and ITDM.

### 2.2. Study Population and Randomization

Patients were eligible for the study when they had an indication for PCI and a suitable angiographic anatomy in a small coronary vessel with a diameter between 2 and 3 mm. Successful predilatation of the lesion, i.e., absence of higher grade dissections (National Heart, Lung, and Blood Institute grade C to F), decreased blood flow (thrombolysis in myocardial infarction score ≤ 2), or residual stenosis > 30% was mandatory [16]. At the time of randomization, diabetes mellitus was defined as history of the disease including ITDM status based on insulin treatment. Exclusion criteria included a concomitant PCI of lesions ≥ 3 mm in diameter in the same epicardial coronary artery, PCI of in-stent restenosis, life expectancy of <12 months, pregnancy, enrollment in another randomized trial, or inability to give informed consent. Patients were randomized 1:1 to be treated by either DCB or DES. 

### 2.3. Procedures

Patients randomized to DCB were treated with the paclitaxel-coated SeQuent Please or SeQuent Please Neo balloon (B. Braun Melsungen AG, Melsungen, Germany), while patients randomized to DES were treated with either the everolimus-eluting Xience stent (Abbott Vascular, Santa Clara, CA, USA) or the paclitaxel-eluting Taxus Element stent (Boston Scientific, Natick, MA, USA) (3, 4, 15). The strut thickness of both DES was 81 μm. The DCB needed to be 2 to 3 mm longer on each side than the predilatation balloon to avoid geographical mismatch, and they were inflated at a nominal pressure for at least 30 s, as recommended in the consensus documents [16]. When there were flow-limiting dissections after DCB treatment despite an acceptable result after lesion preparation, stent implantation was performed. After PCI, DAPT was given using acetylsalicylic acid (100 mg per day) and either clopidogrel (75 mg per day), prasugrel (10 mg per day), or ticagrelor (90 mg twice per day); DAPT was continued in stable patients for 4 weeks for DCB or 6 months for DES and in patients with acute coronary syndromes for 12 months. Follow-up was conducted after 12, 24 and 36 months with structured clinical questionnaires or phone calls to assess clinical events and medication. Patients were followed for a median of 3 years.

### 2.4. Outcomes

The primary endpoint is major adverse cardiac events (MACE) defined as the composite of cardiac death, non-fatal myocardial infarction, and target vessel revascularization (TVR). Cardiac death was defined as any death without a clear cardiac reason, and myocardial infarction was defined according to guidelines [17]. Secondary endpoints are the single components of the primary endpoint according to the Academic Research Consortium definition [18]. An independent critical events committee adjudicated all endpoints.

### 2.5. Statistical Analysis

All statistical analyses were performed according to the intention-to-treat principle, i.e., all patients were analyzed on the basis of the treatment they were randomly allocated to. All analyses were conducted with the statistical software package R, using “two-sided” statistical tests and confidence intervals, without correction for multiple testing. Categorical data are presented as frequencies and percentages with the difference between study arms analyzed by Pearson’s chi-squared test. For numerical variables, the mean and standard deviation, or the median and interquartile range, are presented as appropriate, with the difference between study arms analyzed using Student’s *t*-test or the Wilcoxon–Mann–Whitney test, respectively. For each endpoint, treatment effects on the times to event were tested by Cox regressions (with study center as a stratifying factor to account for differences in baseline hazards between study centers). The Kaplan–Meier estimates of the event rates in both study arms are reported along with the corresponding hazard ratios (HR) and 95% confidence intervals (CI). The proportional hazards assumption of the Cox models and the homogeneity of the treatment effects among study centers were checked by testing the correlation of the scaled Schoenfeld residuals with time and the interaction of the stratifying factor study center with treatment in the Cox models, respectively. Endpoints of patients not experiencing an event were considered as censored on the last observation date.

## 3. Results

Out of the 758 randomized patients, 252 (33.2%) were diabetic and 506 (66.8%) non-diabetic. In the diabetic subgroup, there were 95 (37.7%) patients with ITDM and 157 (62.3%) patients with NITDM. TVR was significantly lower with DCB versus DES in all diabetic patients (HR 0.41, 95% CI 0.18–0.95, *p* = 0.038).

Baseline characteristics between patients with ITDM or NITDM are depicted in Table 1. ITDM patients compared to NITDM patients had a significantly higher body mass index and a significantly higher frequency of renal dysfunction, while other parameters such as hypercholesterolemia, hypertension, previous MI, previous PCI, or antiplatelet therapy were well balanced between the groups.

Table 2 shows the Kaplan–Meier estimates of event rates between the two study arms (DES versus DCB) within the subgroup of patients with ITDM and NITDM for MACE and each single endpoint at one, two, and three years of follow-up. In patients with ITDM, rates of MACE, cardiac death, non-fatal MI, TVR, and all-cause death were statistically not different between patients treated with DCB or DES for up to three years of follow-up. Event rates for TVR were lower in patients treated with DCB compared to DES at all follow-up timepoints. Based on the 3-year TVR rate, the number needed to treat to prevent one additional TVR would be 18 in ITDM.

In the population with NITDM, the rates of MACE, cardiac death, non-fatal MI, TVR, and all-cause death were statistically not different between patients treated with DCB or DES at one, two and three years of follow-up. In NITDM patients, there was a trend toward a lower TVR rate in patients treated with DCB compared to patients treated with DES at two years (DES versus DCB 11.6% vs. 4.5%, *p* = 0.063) and three years (DES versus DCB 14.5% vs. 8.4%, *p* = 0.057) of follow-up. Based on the 3-year TVR rate, the number needed to treat to prevent one additional TVR would be 16 in NITDM.

**Figure 1:**
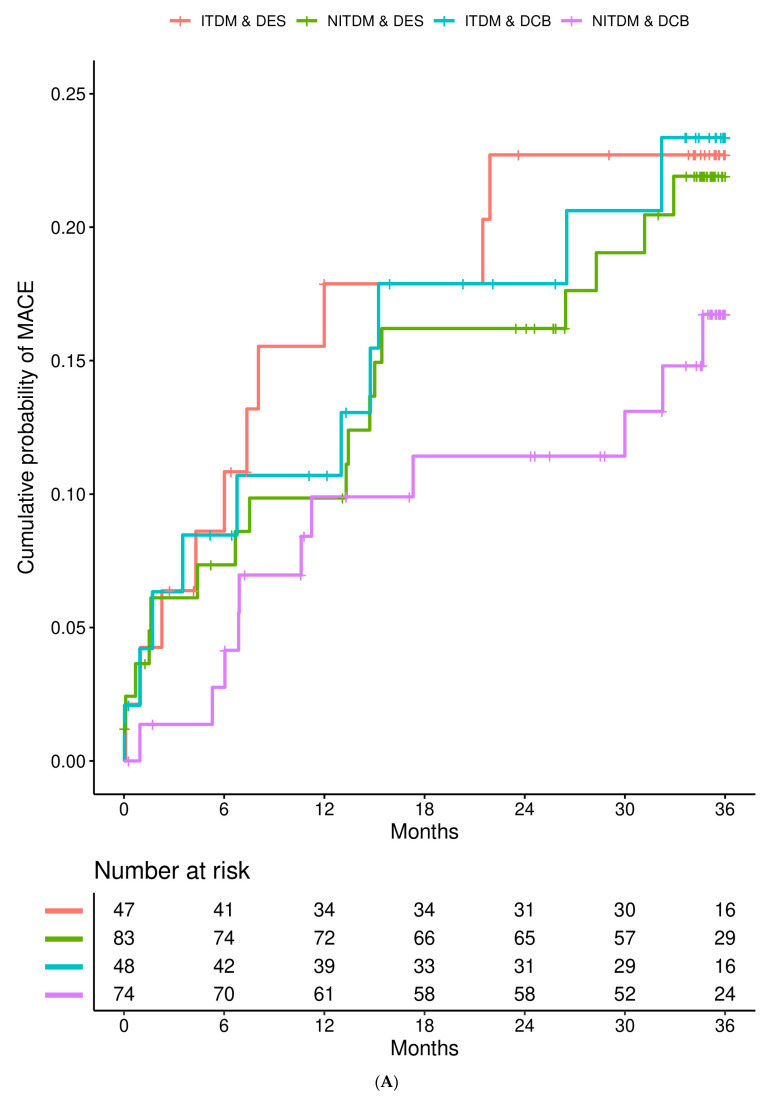

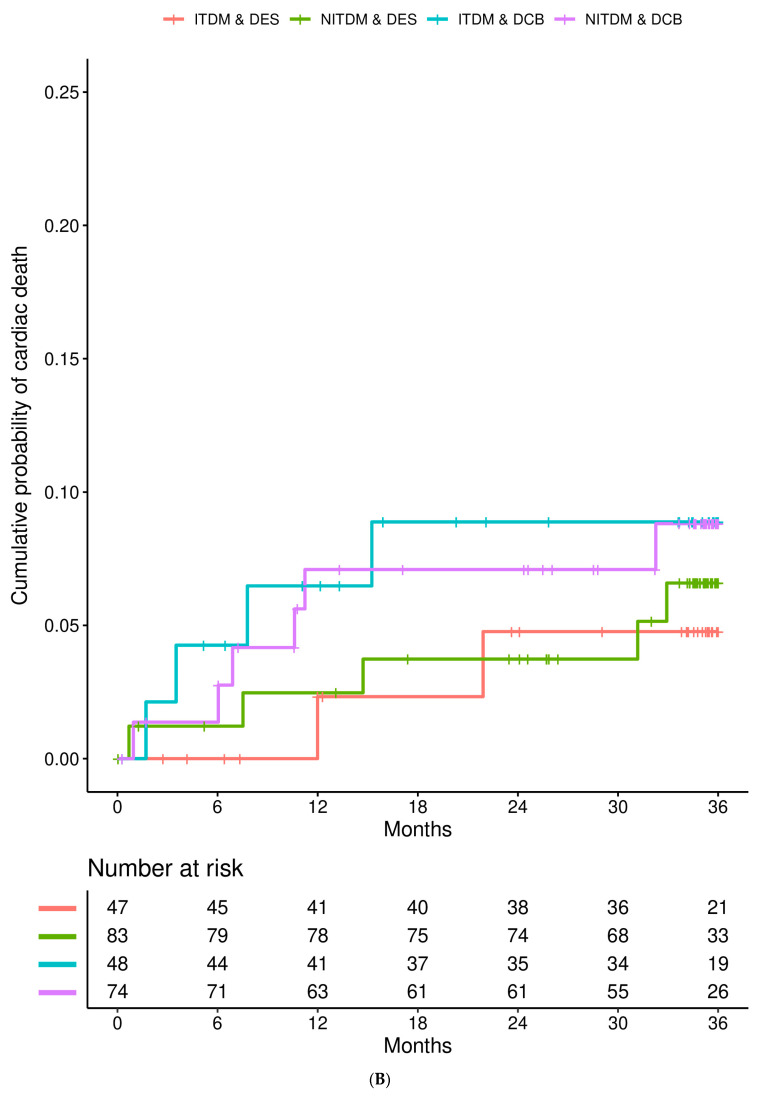

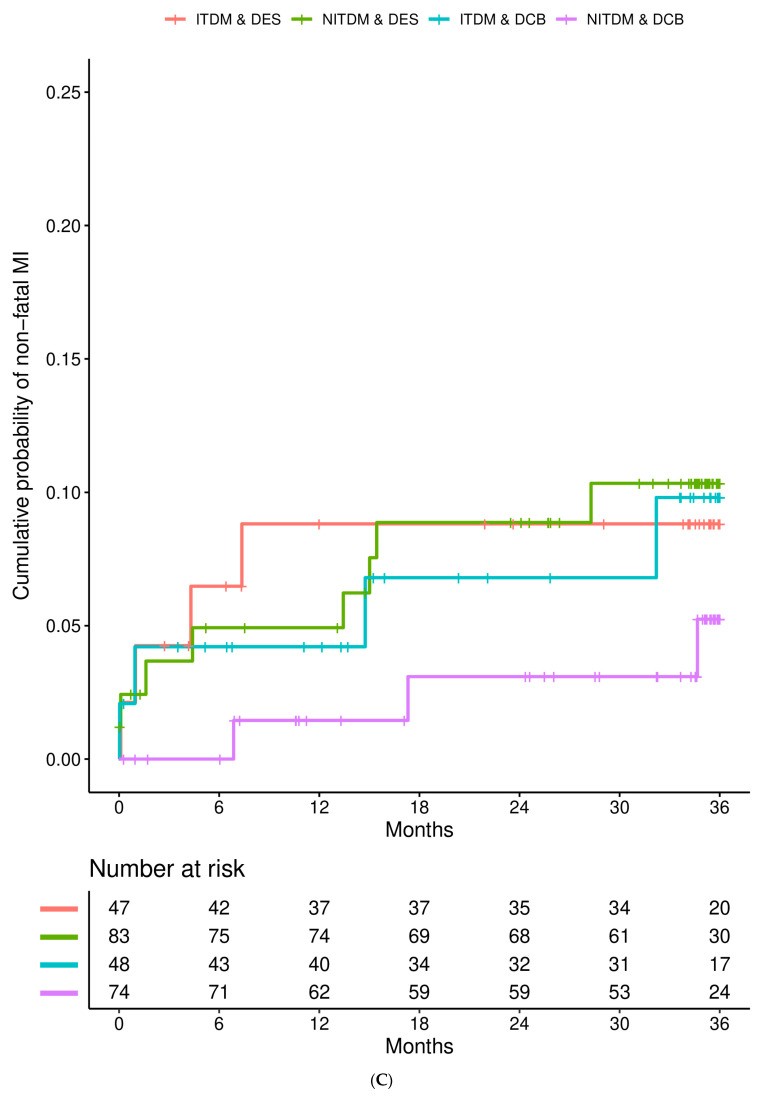

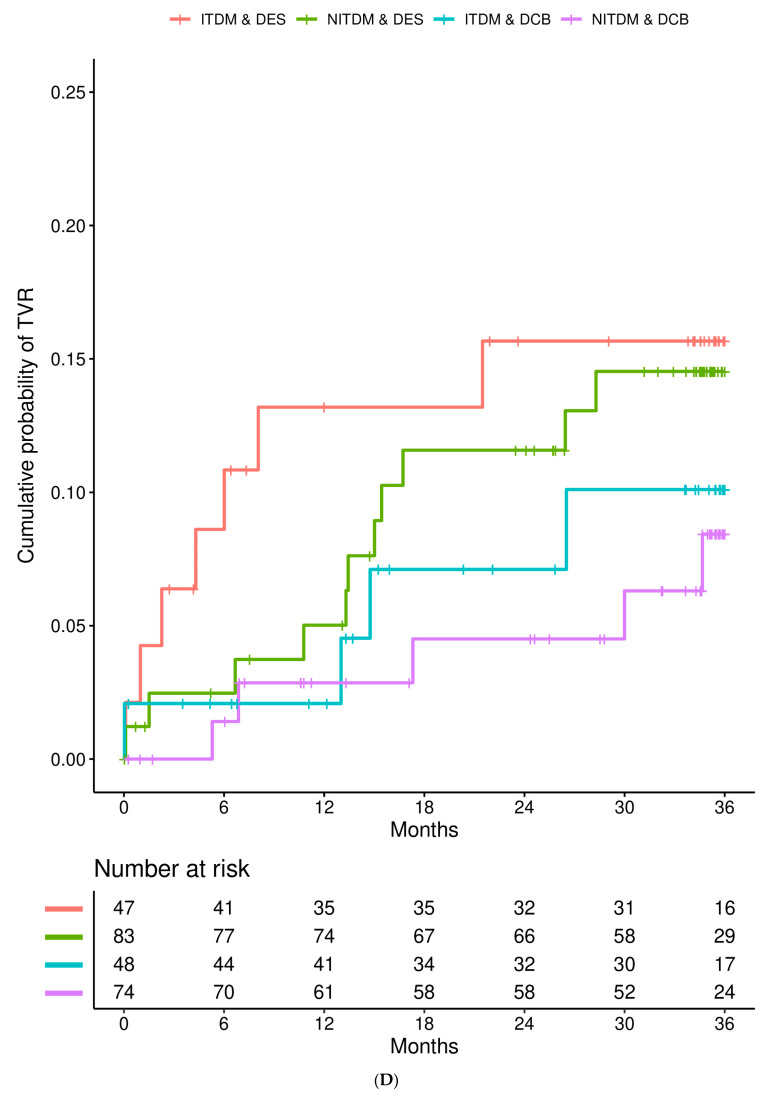
Interaction is presented for diabetes mellitus and randomized treatment strategy. (**A**) Kaplan–Meier estimates of the cumulative probabilities of major adverse cardiac events (MACE) during three years in the four combinations of subgroup and study arm (DES = drug eluting stent, DCB = drug coated balloon, NITDM = non-insulin-treated diabetes mellitus, ITDM = insulin-treated diabetes mellitus); (**B**) Kaplan–Meier estimates of the cumulative probabilities of non-fatal myocardial infarction during three years in the four combinations of subgroup and study arm (DES = drug eluting stent, DCB = drug coated balloon, NITDM = non-insulin-treated diabetes mellitus, ITDM = insulin-treated diabetes mellitus); (**C**) Kaplan–Meier estimates of the cumulative probabilities of target vessel revascularization (TVR) during three years in the four combinations of subgroup and study arm (DES = drug eluting stent, DCB = drug coated balloon, NITDM = non-insulin-treated diabetes mellitus, ITDM = insulin-treated diabetes mellitus); (**D**) Kaplan–Meier estimates of the cumulative probabilities of all-cause death during three years in the four combinations of subgroup and study arm (DES = drug eluting stent, DCB = drug coated balloon, NITDM = non-insulin-treated diabetes mellitus, ITDM = insulin-treated diabetes mellitus).

Figure 1 details the Kaplan–Meier estimates of the cumulative probabilities of MACE (panel A), non-fatal MI (panel B), TVR (panel C), and all-cause death (panel D) during three years in the four combinations of subgroup (ITDM or NITDM) and study arm (DCB or DES). MACE occurred more often in ITDM compared to NITDM patients.

Cox regression analysis stratified by study center with interaction of treatment showed that there was no interaction between ITDM and NITDM and randomized treatment (DCB or DES) with respect to MACE, cardiac death, non-fatal MI, TVR, and all-cause death for all follow-up timepoints (Table 3). In addition, the risk of MACE, non-fatal MI, TVR, and all-cause death was (statistically not significant) lower for patients with NITDM compared to ITDM patients. Risk of TVR was significantly lower with the use of DCB compared to the use of DES after two (0.36 [0.14, 0.94], *p* = 0.037) and three (0.41 [0.18–0.95], *p* = 0.038) years of follow-up.

## 4. Discussion

In this subgroup analysis of the randomized BASKET-SMALL 2 trial, we were able to demonstrate that (1) the risk of MACE, death, non-fatal MI, and TVR is higher in patients with ITDM compared to NITDM for up to three years of follow-up; (2) in patients with ITDM compared to patients with NITDM, rates of MACE, cardiac death, non-fatal MI, TVR, and all-cause death were similar between DCB and DES; and (3) TVR rate was numerically lower with DCB compared to DES both in ITDM and NITDM patients for up to three years of follow-up.

Diabetes, particularly in patients taking insulin, has consistently been shown to be an independent predictor of adverse outcomes after DES. The event rates for diabetic patients on insulin have progressively worse clinical outcomes than the rates for diabetic patients not taking insulin [10,11,12,13,14,19]. Thus, the overall results in analysis of patients with diabetes mellitus strongly depend on the percentage of patients with diabetes on insulin. There is a substantial amount of data comparing the outcome after DES implantation in patients with NITDM versus ITDM, whereas this topic has not been studied in the context of DCB.

For patients treated with DES, a systematic review and meta-analysis of 21,759 ITDM and 15,509 NITDM patients reports significantly higher short and long-term adverse cardiovascular outcomes after PCI in ITDM patients compared with NITDM patients [20]. It is well studied for PCI with DES in diabetic patients that treatment with insulin is associated with a significantly higher target lesion failure rate and higher need for revascularization compared to PCI in diabetic patients with NITDM [10,11,12,13,14,21,22]. At present, there are no data comparing the outcome of DCB versus DES in diabetic patients with de novo coronary artery disease differentiated in NITDM and ITDM. Studies comparing DCB with DES in de novo coronary artery disease in small vessels showed similar results with the use of DCB compared to DES [4,23,24]. In the RESTORE Small Vessel Disease China trial [4], 230 patients with de novo coronary artery disease within a 2.25- and 2.75-mm reference diameter were randomized to DCB or DES in a 1:1 ratio. After 2 years of clinical follow-up, the rate of target lesion failure and target lesion revascularization was similar between the DCB and DES population. Diabetes mellitus was present in 41% of the population, underscoring the importance of diabetes mellitus in patients with de novo lesions in small vessels. Results were not separately given for patients with diabetes mellitus and for patients needing insulin. In a propensity score analysis of 1156 matched patients undergoing PCI with DCB, patients without diabetes mellitus had a significantly lower need for target lesion revascularization during the mean of 366 days of follow-up compared to patients with diabetes mellitus [9]. No results were given with respect to treatment with insulin. In a large registry including 978 patients treated with DCB for de novo coronary artery disease, the presence of diabetes mellitus was associated with a 3.36-fold increased risk of target lesion failure (*p* < 0.002) [25], but results for ITDM or NITDM were not given.

There are several possible advantages with DCB for the treatment of de novo coronary artery disease compared to DES in patients with diabetes mellitus. With DCB, there is no permanent metallic frame or polymer inducing inflammation, neo-atherosclerosis, or triggering neointimal proliferation. There is no long-term risk of stent thrombosis since with DCB nothing is left behind, allowing late lumen enlargement. In addition, patients with diabetes mellitus have an increased risk of bleeding. Use of DCB allows us to shorten the dual antiplatelet treatment compared to DES. We were able to show in the BASKET-SMALL 2 trial that the need for TVR was significantly lower in diabetic patients with the use of DCB compared to DES. In addition, rates of MACE in patients with NITDM and in patients with ITDM were similar with DCB and DES for up to three years of follow-up. The need for TVR was lower with the use of DCB compared to DES both for ITDM and NITDM patients. However, this difference did not reach statistical significance due to the limited number of patients.

Small vessel coronary disease is an independent predictor for poor outcomes after PCI [26]. The use of DES in such lesions is associated with higher rates of stent failure, restenosis, and repeat revascularization [26,27]. Metallic DES prohibit late lumen enlargement and limit the full restoration of vessels’ endothelial functions [28,29]. Since several randomized trials have shown similar results for DCB versus DES in de novo lesions of small coronary arteries, DCB are an effective and safe alternative treatment strategy in those lesions. Similar to the use of DES, the use of DCB in patients with diabetes mellitus is associated with higher rates of adverse events compared to patients without diabetes mellitus. Of note, preliminary data point out that in diabetic patients the use of DCB compared to DES may be beneficial with respect to a lower need for repeat TLR. Similar to the use of DES, the need for insulin in the setting of de novo coronary artery disease in small vessels in diabetic patients treated with DCB is associated with higher adverse events compared to NITDM patients.

## 5. Limitations

In this subgroup analysis of the randomized BASKET-SMALL 2 trial, the number of patients in the ITDM and NITDM populations is limited and does not confer enough power to draw definitive conclusions regarding clinical endpoints. Due to the limited number of patients, potential concerns arising from beta error cannot be ruled out. Our results should be interpreted as hypothesis generating and should be confirmed in a randomized trial including patients with ITDM and NITDM. The use of intracoronary imaging was not integrated in the study protocol. Since patients in the study received treatment with paclitaxel-iopromide-coated DCB, these long-term results can only be extrapolated to those who received this type of DCB since there is no class effect. Data regarding duration of diabetes mellitus, dosage of insulin or other medication, or values of hemoglobin A1C were not captured in the BASKET-SMALL 2 trial.

## 6. Conclusions

Based on the randomized BASKET SMALL 2 trial, the rates of MACE, non-fatal MI, and cardiac death are similar between DCB and DES for patients with ITDM and NITDM. The study demonstrates the sustained efficacy and safety of DCB in diabetic patients with de novo lesions of small coronary vessels for up to 3 years compared to DES both for ITDM and NITDM.

## Figures and Tables

**Table 1:** Baseline Characteristics.

	Overall	ITDM	NITDM	*p*-Value
*n*	252	95	157	
age	69.9 (9.5)	70.9 (8.5)	69.3 (9.9)	0.1678
sex = male	178 (70.6)	56 (58.9)	122 (77.7)	0.0025
BMI	29.8 (5.0)	31.1 (5.2)	29.0 (4.7)	0.0012
current smoker	34 (13.5)	10 (10.5)	24 (15.2)	0.5407
former smoker	98 (43.6)	39 (41.1)	59 (37.6)	
no	116 (46.0)	45 (47.4)	71 (45.2)	
hypercholesterolemia	187 (74.2)	75 (78.9)	112 (71.3)	0.2663
hypertension	232 (92.1)	91 (95.8)	141 (89.8)	0.1438
family history	93 (36.9)	34 (35.8)	59 (37.6)	0.5411
prev. anterior MI	46 (18.3)	19 (20.0)	27 (17.2)	0.6966
prev. other MI	58 (23.0)	22 (23.2)	36 (22.9)	1.0000
prev. any MI	99 (39.3)	39 (41.1)	60 (38.2)	0.7538
prev. PCI	160 (63.5)	67 (70.5)	93 (59.2)	0.0951
prev. CABG	28 (11.1)	13 (13.7)	15 (9.6)	0.4213
heart failure	41 (16.3)	16 (16.8)	25 (15.9)	0.9877
stroke	16 (6.3)	8 (8.4)	8 (5.1)	0.4601
TIA	11 (4.4)	3 (3.2)	8 (5.1)	
no	225 (89.3)	84 (88.4)	141 (89.8)	
aortic aneurysm	2 (0.8)	2 (2.1)	0 (0.0)	0.2745
PAOD	21 (8.3)	8 (8.4)	13 (8.3)	1.0000
COPD	26 (10.3)	9 (9.5)	17 (10.8)	0.8975
STEMI	5 (2.0)	3 (3.2)	2 (1.3)	0.6404
NSTEMI	35 (13.9)	15 (15.8)	20 (12.7)	
unstable	26 (10.3)	10 (10.5)	16 (10.2)	
stable	186 (73.8)	67 (70.5)	119 (75.8)	
acute coronary disease	66 (26.2)	28 (29.5)	38 (24.2)	0.4388
liver disease	12 (4.8)	5 (5.3)	7 (4.5)	1.0000
rheumatologic disorder	9 (3.6)	5 (5.3)	4 (2.5)	0.4381
renal dysfunction	82 (32.5)	41 (43.2)	41 (26.1)	0.0078
coronary LM	8 (3.2)	5 (5.3)	3 (1.9)	0.2712
coronary LAD	214 (84.9)	87 (91.6)	127 (80.9)	0.0343
coronary LCX	195 (77.4)	76 (80.0)	119 (75.8)	0.5368
coronary RCA	163 (64.7)	60 (63.2)	103 (65.6)	0.7965
multi-vessel coronary disease	204 (81.0)	80 (84.2)	124 (79.0)	0.3903
ejection fraction %	60.0 [50.0, 60.0]	60.0 [50.0, 61.3]	60.0 [49.3, 60.0]	0.7926
prev. clopidogrel	65 (25.8)	29 (30.5)	36 (22.9)	0.2351
prev. ASS	198 (78.6)	79 (83.2)	119 (75.8)	0.2218
prev. prasugrel	18 (7.1)	6 (6.3)	12 (7.6)	0.8853
prev. ticagrelor	38 (15.1)	10 (10.5)	28 (17.8)	0.1647
prev. statin	176 (69.8)	72 (75.8)	104 (66.2)	0.1647
prev. anticoagulants	26 (10.3)	6 (6.3)	20 (12.7)	0.1641

Categorical variables are depicted as frequencies and percentages and numerical variables as mean and standard deviation (except for ejection fraction as median and interquartile range); *p*-values for the difference between study arms obtained by Pearson’s chi-squared test and Student’s *t*-test, respectively, are given (Wilcoxon–Mann–Whitney test for ejection fraction).

**Table 2:** Comparison of event numbers and Kaplan–Meier estimates of event rates between the two study arms within each subgroup for all endpoints.

Type of Event	Subgroup	Study Arm	1-y Events	1-y HR [95% CI]	2-y Events	2-y HR [95% CI]	3-y Events	3-y HR [95% CI]
MACE	ITDM	DES DCB	8 (17.88%) 5 (10.70%)	1—reference— 0.75 [0.24, 2.35] (*p* = 0.621)	10 (22.71%) 8 (17.88%)	1—reference – 0.89 [0.34, 2.31] (*p* = 0.813)	10 (22.71%) 10 (23.36%)	1—reference— 1.12 [0.46, 2.74] (*p* = 0.808)
MACE	NITDM	DES DCB	8 (9.85%) 7 (9.90%)	1—reference— 0.99 [0.32, 3.06] (*p* = 0.990]	13 (16.20%) 8 (11.43%)	1—reference— 0.70 [0.27, 1.85] (*p* = 0.476)	17 (21.91%) 11 (16.74%)	1—reference— 0.68 [0.29, 1.58] (*p* = 0.372)
cardiac death	ITDM	DES DCB	1 (2.33%) 3 (6.48%)	1—reference— 3.42 [0.35, 33.39] (*p* = 0.290)	2 (4.77%) 4 (8.88%)	1—reference— 2.25 [0.41, 12.37] (*p* = 0.353)	2 (4.77%) 4 (8.88%)	1—reference— 2.25 [0.41, 12.37] (*p* = 0.353)
cardiac death	NITDM	DES DCB	2 (3.47%) 5 (7.09%)	1—reference— 5.38 [1.02, 28.32] (*p* = 0.047)	3 (3.74%) 5 (7.09%)	1—reference— 3.47 [0.81,14.80] (*p* = 0.093)	5 (6.59%) 6 (8.82%)	1—reference— 2.47 [0.73, 8.34] (*p* = 0.145)
non-fatal MI	ITDM	DES DCB	4 (8.82%) 2 (4.21%)	1—reference— 0.73 [0.13, 4.27] (*p* = 0.730)	4 (8.82%) 2 (6.80%)	1—reference— 1.00 [0.21, 4.80] (*p* = 0.999)	4 (8.82%) 4 (9.81%)	1—reference— 1.36 [0.32, 5.78] (*p* = 0.679)
non-fatal MI	NITDM	DES DCB	4 (4.93%) 1 (1.45%)	1—reference— 0.17 [0.02, 1.54] (*p* = 0.115)	7 (8.87%) 2 (3.09%)	1—reference— 0.10 [0.01, 1.00] (*p* = 0.050)	8 (10.34%) 3 (5.25%)	1—reference— 0.21 [0.04, 1.12] (*p* = 0.068)
TVR	ITDM	DES DCB	6 (13.19%) 1 (2.08%)	1—reference— 0.21 [0.02, 1.81] (*p* = 0.156)	7 (15.67%) 3 (7.11%)	1—reference— 0.48 [0.12, 1.92] (*p* = 0.297)	7 (15.67%) 4 (10.11%)	1—reference— 0.64 [0.18, 2.279. (*p* = 0.489)
TVR	NITDM	DES DCB	4 (5.02%) 2 (2.86%)	1—reference— 0.37 [0.06, 2.41] (*p* = 0.299)	9 (11.58%) 3 (4.50%)	1—reference— 0.25 [0.06, 1.08] (*p* = 0.063)	11 (14.53%) 5 (8.44%)	1—reference— 0.30 [0.09, 1.03] (*p* = 0.057)
all-causes death	ITDM	DES DCB	2 (4.45%) 4 (8.56%)	1—reference— 2.26 [0.41, 12.45] (*p* = 0.350)	4 (9.11%) 7 (15.72%)	1—reference— 2.19 [0.63, 7.60] (*p* = 0.217)	5 (11.50%) 7 (15.72%)	1—reference— 1.71 [0.53, 5.46] (*p* = 0.366)
all-causes death	NITDM	DES DCB	3 (3.67%) 7 (9.75%)	1—reference— 4.23 [1.05, 17.11] (*p* = 0.043)	6 (7.38%) 8 (11.21%)	1—reference— 1.79 [0.56, 5.70] (*p* = 0.324)	10 (12.64%) 10 (14.41%)	1—reference— 1.43 [0.55, 3.74] (*p* = 0.466)

Cox regression stratified by study center, MACE = major adverse cardiac events, MI = myocardial infarction, TVR = target vessel revascularization, HR = hazard ratio, CI = confidence intervals. Rates are Kaplan–Meier rates at each landmark time.

**Table 3:** Cox regressions stratified by study center with and without interaction with treatment.

Type of Event	Variable	1-y HR [95% CI] (*p*-Value)	2-y HR [95% CI] (*p*-Value)	3-y HR [95% CI] (*p*-Value)
MACE	study arm: DCB vs. DES subgroup: NITDM vs. ITDM	0.82 [0.38, 1.76] (*p* = 0.604) 0.59 [0.27, 1.28 0.(*p* = 0.183)	0.77 [0.40, 1.49] (*p* = 0.442) 0.63 [0.33, 1.21] (*p* = 0.169)	0.81 [0.45, 1.47] (*p* = 0.493) 0.75 [0.42, 1.36] (*p* = 0.348)
MACE	study arm: DCB vs. DES subgroup: NITDM vs. ITDM interaction: NITDM and DCB	0.85 [0.39, 1.87] (*p* = 0.688) 0.61 [0.28, 1.33] (*p* = 0.215) 1.48 [0.31, 704] (*p* = 0.623)	0.76 [0.39, 1.49] (*p* = 0.422) 0.63 [0.32, 1.21] (*p* = 0.163) 0.85 [0.23, 3.18] (*p* = 0.805)	0.78 [0.43, 1.44] (*p* = 0.431) 0.73 [0.40, 1.34] (*p* = 0.314) 0.63 [0.19, 2.13] (*p* = 0.457)
cardiac death	study arm: DCB vs. DES subgroup: NITDM vs. ITDM	3.93 [1.03, 15.04] (*p* = 0.046) 1.16 [0.33, 4.13] (*p* = 0.813)	2.46 [0.82, 7.43] (*p* = 0.110) 0.89 [0.30, 2.63] (*p* = 0.830)	2.06 [0.77, 5.50] (*p* = 0.150) 1.24 [0.45, 3.43] (*p* = 0.678)
cardiac death	study arm: DCB vs. DES subgroup: NITDM vs. ITDM interaction: NITDM and DCB	3.85 [1.01, 14.63] (*p* = 0.048) 1.05 [0.25, 4.41] (*p* = 0.948) 1.50 [0.09, 25.19] (*p* = 0.779)	2.49 [0.83, 7.49] (*p* = 0.105) 0.84 [0.27, 2.63] (*p* = 0.769) 1.37 [0.14, 12.90] (*p* = 0.786)	2.06 [0.77, 5.50] (*p* = 0.151) 1.24 [0.43, 3.56] (*p* = 0.695) 1.03 [0.13, 8.39] (*p* = 0.977)
non-fatal MI	study arm: DCB vs. DES subgroup: NITDM vs. ITDM	0.32 [0.08, 1.32] (*p* = 0.116) 0.42 [0.12, 1.44] (*p* = 167)	0.45 [0.14, 1.37] (*p* = 0.157) 0.67 [0.24, 1.86] (*p* = 0.441)	0.56 [0.21, 1.49] (*p* = 0.242) 0.75 [0.29, 1.91] (*p* = 0.546)
non-fatal MI	study arm: DCB vs. DES subgroup: NITDM vs. ITDM interaction: NITDM and DCB	0.24 [0.05, 1.18] (*p* = 0.079) 0.30 [0.08, 1.24] (*p* = 0.096) 0.22 [0.01, 4.04] (*p* = 0.309)	0.37 [0.11, 1.24] (*p* = 0.106) 0.52 [0.17, 1.58] (*p* = 0.248) 0.23 [0.02, 2.30] (*p* = 0.212)	0.49 [0.17, 1.40] (*p* = 0.181) 0.65 [0.24, 1.75] (*p* = 0.392) 0.29 [0.04, 2.22] (*p* = 0.234)
TVR	study arm: DCB vs. DES subgroup: NITDM vs. ITDM	0.28 [0.08, 1.04] (*p* = 0.058) 0.50 [0.16, 1.54] (*p* = 0.227)	0.36 [0.14, 0.94] (*p* = 0.037) 0.67 [0.28, 1.60] (*p* = 0.368)	0.41 [0.18, 0.95] (*p* = 0.038) 0.78 [0.35, 1.72] (*p* = 0.539)
TVR	study arm: DCB vs. DES subgroup: NITDM vs. ITDM interaction: NITDM and DCB	0.30 [0.08, 1.16] (*p* = 0.081) 0.62 [0.16, 2.48] (*p* = 0.500) 2.28 [0.14, 36.22] (*p* = 0.560)	0.34 [0.13, 0.93] (*p* = 0.035) 0.61 [0.23, 1.61] (*p* = 0.320) 0.66 [0.10, 4.57] (*p* = 0.673)	0.39 [0.17, 0.92] (*p* = 0.032) 0.71 [0.31, 1.63] (*p* = 0.418) 0.55 [0.10, 3.09] (*p* = 0.493)
all-causes death	study arm: DCB vs. DES subgroup: NITDM vs. ITDM	2.91 [0.99, 8.56] (*p* = 0.052) 0.99 [0.35, 2.80] (*p* = 0.981)	1.71 [0.75, 3.88] (*p* = 0.201) 0.70 [0.31, 1.59] (*p* = 0.400)	1.40 [0.68, 2.88] (*p* = 0.355) 0.92 [0.44, 1.93] (*p* = 0.832)
all-causes death	study arm: DCB vs. DES subgroup: NITDM vs. ITDM interaction: NITDM and DCB	2.91 [1.00, 8.50] (*p* = 0.051) 0.88 [0.29, 2.68] (*p* = 0.822) 1.78 [0.20, 16.12] (*p* = 0.609)	1.68 [0.73, 3.87] (*p* = 0.220) 0.72 [0.31, 1.66] (*p* = 0.437) 0.83 [0.16, 4.40] (*p* = 0.827)	1.40 [0.68, 2.88] (*p* = 0.360) 0.93 [0.44, 1.95] (*p* = 0.846) 0.88 [0.20, 3.92] (*p* = 0.870)

## Data Availability

Not applicable.
